# Investigations on Stub-Based UWB-MIMO Antennas to Enhance Isolation Using Characteristic Mode Analysis

**DOI:** 10.3390/mi13122088

**Published:** 2022-11-27

**Authors:** Ankireddy Chandra Suresh, Thatiparthi Sreenivasulu Reddy, Boddapati Taraka Phani Madhav, Sudipta Das, Sunil Lavadiya, Abeer D. Algarni, Walid El-Shafai

**Affiliations:** 1 Department of Electronics and Communication Engineering, Sri Venkateswara University College of Engineering, SV University, Tirupathi 517502, A.P, India; 2 Antennas and Liquid Crystals Research Center, Department of Electronics and Communication Engineering, Koneru Lakshmaiah Education Foundation, Vaddeswaram 522303, India; 3 Department of Electronics & Communication Engineering, IMPS College of Engineering and Technology, Malda 732103, India; 4 Department of Information and Communication Technology, Marwadi University, Rajkot 360003, India; 5 Department of Information Technology, College of Computer and Information Sciences, Princess Nourah bint Abdulrahman University, P.O. Box 84428, Riyadh 11671, Saudi Arabia; 6 Security Engineering Lab, Computer Science Department, Prince Sultan University, Riyadh 11586, Saudi Arabia; 7 Department of Electronics and Electrical Communications Engineering, Faculty of Electronic Engineering, Menoufia University, Menouf 32952, Egypt

**Keywords:** CMA, decoupling stub, ECC, isolation, UWB-MIMO

## Abstract

In this article, very compact 2 × 2 and 4 × 4 MIMO (Multiple-Input and Multiple output) antennas are designed with the help of Characteristics Mode Analysis to enhance isolation between the elements for UWB applications. The proposed antennas are designed with Characteristic Mode Analysis (CMA) to gain physical insight and also to analyze the dominant mode. To improve isolation and minimize the mutual coupling between radiating elements, elliptical shaped stubs are used. The dimensions of the 2 × 2 and 4 × 4 MIMO antennas are 0.29λ_0_ × 0.21λ_0_ (28 × 20 mm^2^) and 0.29λ_0_ × 0.42λ_0_ (28 × 40 mm^2^), respectively. These antennas cover the (3.1 GHz–13.75 GHz) UWB frequency band and maintain remarkable isolation of more than 25 dB for both 2 × 2 and 4 × 4 antennas. The impedance bandwidth of the proposed 4 × 4 MIMO antenna is 126.40% from 3.1 GHz to 13.75 GHz, including X-Band and ITU bands. The proposed 4 × 4 antenna has good radiation efficiency, with a value of more than 92.5%. The envelope correlation coefficient (ECC), diversity gain (DG), mean effective gain (MEG), and channel capacity loss (CCL) matrices of the 4 × 4 antenna are simulated and tested. The corresponding values are 0.0045, 9.982, −3.1 dB, and 0.39, respectively. The simulated results are validated with measured results and favorable agreements for both the 2 × 2 and 4 × 4 UWB-MIMO antennas.

## 1. Introduction

The release of the unlicensed frequency spectrum from 3.1 GHz–10.6 GHz (UWB) by the Federal Communications Commission resulted in significant changes in wireless communications, including high-speed transmission, data rate, and higher security. But the problems faced by UWB are fading, multipath environments, and low radiating powers of less than 41.3 dB/MHz [1]. These drawbacks of UWB technology reduce its performance. In the future, high data rates will be required with more than 100 Gbps of bandwidth to meet all wireless communications applications. The reliable technology called MIMO (Multiple Input and Multiple Output) increases the channel capacity, resulting in high data rates and high speeds of data transmission. The MIMO maximizes the reduction in multiple paths and fading problems so that the combined UWB-MIMO technology attains better link quality and higher data rates. However, the challenge faced by UWB-MIMO is the size of the antennas. Several MIMO elements are integrated into a small space, producing high mutual coupling and correlation problems. Therefore, several methods have been adopted to enhance isolation and reduce mutual couplings, including introducing slots, placing stubs, and Electromagnetic Band-Gap (EBG) in between MIMO elements and also placing conducting materials called parasitic materials in between MIMO elements. Spatial and pattern diversity techniques are also used to improve isolation in MIMO elements [2]. The periodical arrangements of Complimentary Splint Ring Resonator (CSRR) metal strips or capacitive gaps perform filtering action and enhance isolation between MIMO elements [3].

In the UWB antenna with dimensions 100 × 150 mm^2^, a metasurface-based decoupling method (MDM) was used, and antenna elements were composed of the metasurface’s superstrate to achieve isolation greater than 25 dB [4]. In the Fractal MIMO antenna, an L-shaped inverted slot was used to reduce the mutual coupling up to 34 dB [5,6]. The half-hexagonal-shaped monopoles included grounded circular rings along with grounded stubs that improved the isolation by 20 dB [7]. Several decoupling methods are used to maximize isolation [8,9,10,11,12]. A very high-compact four-port vertical polarized UWB MIMO antenna covered a 2.2 GHz–12.3 GHz band with high isolation of more than 15 dB. A Specific interlocking decoupling method was used to achieve low mutual coupling [13]. A very sophisticated mutual coupling technique is called “neutralizing lines,” which were added into 2 × 2 and 4 × 4 very compact MIMO elements to enhance isolation, −22 dB, and achieve a low CCL of less than 0.29 bits/Hz [14]. Pentagonal MIMO antenna structures were investigated to achieve high isolation of more than 28 dB by inserting slots and plus-shaped parasitic structure between the patch elements [15]. 

A miniaturized 4 × 4 UWB MIMO antenna has been designed with dimensions of 40 × 40 × 1.567 mm^3^. This antenna is very simple in structure and covers UWB (3.1 GHz-10.6 GHz) and ITU bands (3.1 GHz–13.25 GHz), with high isolation of more than 15 dB, and the achieved radiation efficiency is 89% [16]. A rectangular microstrip stub with defected ground plane is employed to increase the isolation >15 dBfor the reported dual band MIMO antenna [17]. However, the above work provides only partial isolation improvement techniques, because the design process lacks a systematic approach, so it does not meet the quality requirement of antenna design. A systematic design approach is required to understand the physical insight of the antenna structure, which gives immense knowledge of the physical structure to antenna designers and allows a good design process of the antenna in less time with more sophisticated results. The generated currents and scattered fields from perfect electric conductors are explained by CMC [18,19]. 

The mode currents are the sum of orthogonal currents from PEC bodies, and modes have orthogonal characteristics across the surface [20]. The various advantages of CMA are used to design sophisticated antennas in a systematic design approach. The CMA metrics and modal significance provides the bandwidth, coupling capability, and insight into the exciting mode of the radiating element without exciting the feed [21]. The performance of multiple antennas in a systematic design approach is achieved by Characteristic Mode Analysis [22]. Two metal strips were combined to form the chassis to generate two characteristic modes with wideband properties, and to achieve high isolation [23,24]. The diversity radiation pattern and high isolation are achieved by two monopole antennas. One radiator is the compact quarter loop and the second one is a circular planar monopole. Both are ultra-wide antennas located at opposite faces of the rectangular ground plane of MIMO, and both elements are excited in different modes [25]. By using the symmetry of the etched slots, the required isolation of more than 32 dB is achieved between antenna elements [26]. A 4 × 4 reconfigurable UWB MIMO antenna is designed to achieve isolation greater than 20 dB in an entire band of 3 GHz-11 GHz. This antenna shows some band rejection characteristics due to the placing of the PIN diode. Different geometry shapes and novel decoupling techniques were also used to achieve high isolation, good radiation performance, and improved diversity [27,28,29,30]

The majority of the preceding work employed orthogonal modes and defected ground structures to improve isolation in MIMO antennas, and some antennas employed space diversity techniques. The above works need a more systematic design approach. 

In this work, a new systematic methodology is proposed to achieve improved isolation in MIMO antenna using Characteristic Mode Analysis. The elements in the proposed antenna, 2 × 2 and 4 × 4 UWB-MIMO, are placed symmetrically over the FR-4 substrate to achieve high compactness.

### Novelty and Contributions

The main important points focused on while designing the 2 × 2 and 4 × 4 MIMO antennas are:

1. The proposed compact 2 × 2 and 4 × 4 UWB-MIMO antennas achieved −25 dB isolation among elements using Characteristic Mode Analysis (CMA).
2. The designed antennas not only cover UWB but also cover the X and ITU bands.
3. The decoupling I-shaped stub is placed at ground level to enhance the isolation and overall performance of the MIMO antennas.
4. A step-by-step systematic designing approach using CMA methodology is used to achieve good diversity characteristics in operating frequency range.
5. Both 2 × 2 and 4 × 4 antennas achieve good gain and radiation efficiencies.
6. The designed 2 × 2 UWB-MIMO achieved a peak gain and radiation efficiency of 4.2 dB and 82.5%, respectively. Therefore, there is a scope to enhance the above said parameters by changing the 2 × 2 configuration into a 4 × 4 MIMO arrangement.
7. The proposed 4 × 4 UWB-MIMO with CMA achieved gain and radiation efficiency up to 4.8 dB and 92.2%, respectively. The ECC of 2 × 2 is also reduced from 0.005 to 0.0045 by designing 4 × 4 MIMO antenna.

The rest of the paper is organized as follows. The detailed design of 2 × 2 with CMA is explained in Section 2. Section 3 explains the result of 2 × 2 MIMO antenna. Section 4 is organized to explain the 4 × 4 UWB-MIMO antenna. Performance characteristics and results of 4 × 4 antenna are analyzed in Section 5, and in Section 6 comparisons with existing models are discussed. Finally, this paper ends with conclusions in Section 7.

## 2. Antenna Design

### 2.1. UWB-MIMO Using CMA

The designed 2 × 2 antenna is composed of two symmetrical circular radiators at the top, each having rectangular discs that behave in a similar way to radiating elements. The ground plane is composed of two elements: (i) partial ground plane and (ii) elliptical shape decoupling stub. 

**Figure 1:**
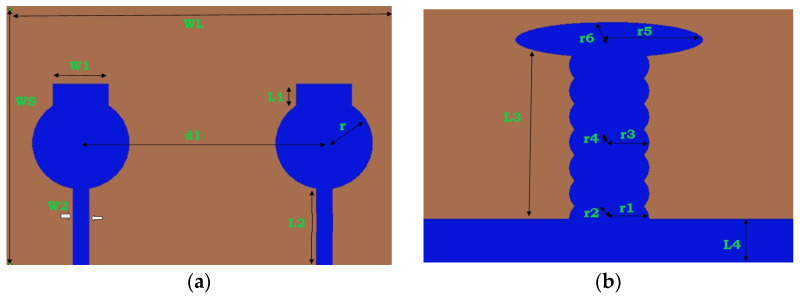
The dimensions of the proposed high-compact 2 × 2 UWB MIMO antenna. (**a**) Top view, (**b**) Bottom view.

The overall dimensions of the proposed 2 × 2 antenna are 0.29λ_0_ × 0.21λ_0_ (28 × 20 mm^2^) printed on an FR-4 substrate, with tanẟ = 0.002 and $\varepsilon_{r}\;$= 4.3, as depicted in Figure 1. To improve low-frequency isolation, this proposed antenna uses a circular patch and a rectangular disc. The designed parameters with dimensions are listed in Table 1.

The radius (*r*) of 2 × 2 circular Patch radiator [31] is determined by using the following expressions:

(1) $$r=\frac{F}{\sqrt{1+\frac{2h}{\pi \epsilon_{r}F}\left[\ln\left(\frac{\pi F}{2h}\right)+1.7726\right]}}\;$$

where *F* is given by:

(2) $$F=\frac{8.791\times 10^{9}}{f_{r}\sqrt{\epsilon_{r}}}\;$$

The effective radius results from the fringing field spreading from the patch border to the ground plane. The fringing field around the circular patch may increase the radius of a circle. Therefore, the effective radius (*r_eff_*) is determined as shown below:

(3) $$\;r_{eff}=\frac{1.8412\times c}{2\pi f_{r}\sqrt{\epsilon_{r}}}\;$$

where *c* is velocity of light in vacuum = 3 × 10^11^ mm/s, and *f_r_* is resonant frequency. The dielectric material FR-4 is used, its height is *h* = 1.6 mm, and its operating frequency is between 3.1 GHz and 13.75 GHz. This proposed antenna well resonates in the UWB range. The specifications are$\;\epsilon_{r}$ = 4.3 and *h* = 0.16 cm, and *f_r_* = 7.5 GHz is used for calculating the radius of the patch.

$$r=\frac{F}{\sqrt{1+\frac{2h}{\pi \epsilon_{r}F}\left[\ln\left(\frac{\pi F}{2h}\right)+1.7726\right]}}=\frac{0.6}{\sqrt{1+\frac{2\times 0.16}{\pi \times 4.3\times 0.6}\left[\ln\left(\frac{\pi \times 0.6}{2\times 0.16}\right)+1.7726\right]}}=3.6\;\mathrm{mm}$$

The radius (*r*) = 3.6 mm is suitable for resonating the antenna in UWB band.

**Figure 2:**
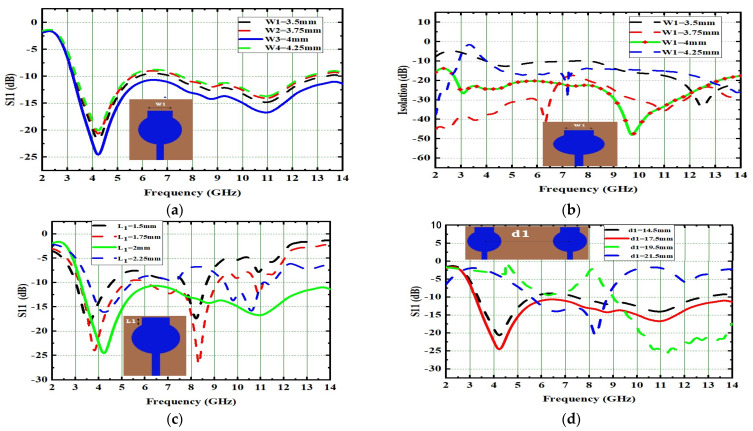
Parametric variations of single radiator w.r.t. (**a**) Width of the of the horizontal disc W1, (**b**) Isolation due to variations in W1, (**c**) Variations in L1 (Length of rectangular disc L1), (**d**) Variations in d1 (Distance between two radiators d1).

The reflection coefficient of the single radiator is initially analyzed by changing the horizontal stub (W1) from 3.5 mm to 4.25 mm. The corresponding reflection coefficient and isolation are depicted in Figure 2a,b. The same procedure is repeated by varying the values of the Length of the rectangular disc (L1) and the distance between two radiating elements (d1), and its corresponding reflection coefficients are depicted in Figure 2c,d.

The horizontal disc’s width (W1) was adjusted from 3.5 mm to 4.25 mm and the current distributions were simultaneously observed. The current distributions were excellent at low frequencies when W1 = 3.5 mm, but the UWB range was not covered. The current distributions were excellent at high frequencies (>10.6 GHz) at W1 = 4.25 mm. However, at lower frequencies and in the UWB range they were not appreciable. The current distributions shifted from the low-frequency to the middle-frequency area at W1 = 4 mm. Thus, at W1 = 4 mm, the designed MIMO effectively spanned the impedance bandwidth. Therefore, the antenna provides good impedance at W1 = 4 mm and, as a result, good current distribution in the UWB. The horizontal disc’s length (L1) was adjusted from 1.5 to 2.25 mm, and the current distribution was observed. The current distribution was excellent at lower frequencies when L1 was 1.5 mm. However, it was not impressive in the UWB and higher frequency regions. When the L1 was changed from 1.5 mm to 2.25 mm, the current distribution over the antenna surface shifted in the middle portion from the low-frequency region to the high-frequency region and did not cover the UWB range. The maximum current distributions shifted from the low-frequency to the middle-frequency area at L1 = 2 mm, so that at L1 = 2 mm, this MIMO adequately covered the good impedance bandwidth’s entire UWB region. 

The isolation between the MIMO elements has to be maintained, otherwise the interaction between the elements would increase and the MIMO diversity characteristics may not meet. The space diversity technique can be used to improve the isolation. The current distribution interaction between MIMO elements can be reduced by using additional space between the MIMO elements, but this technique needs more space. The distance between elements (d1) in this MIMO design ranged from 14.5 mm to 21.5 mm. Low mutual coupling between the MIMO components was not achieved for the element spacing of d1 = 14.5 mm. The mutual coupling between the elements is weak at d1 = 21.5 mm, but the antenna size increases. The selected value of d1 = 17.5 mm obtained good isolation among the antenna elements for the MIMO’s compactness.

Antenna 0 (A#0), Antenna 1(A#1), Antenna 2(A#2), and Antenna 3(A#3) are the four stages of the design evolution process. The proposed highly compacted 2 × 2 antenna can be transformed into a 4 × 4 MIMO antenna without the need for additional design methods.

**Figure 3:**
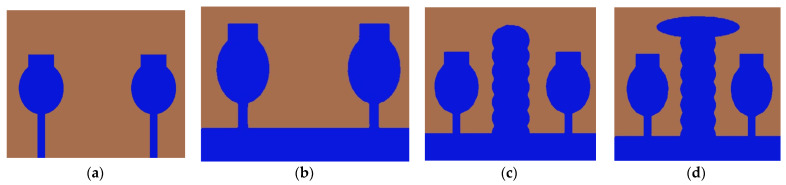
Evolution of 2 × 2 UWB MIMO antenna. (**a**) A#0, (**b**) A#1, (**c**) A#2, (**d**) A#3.

The design procedure of 2 × 2 is performed by Characteristic Mode Analysis. The physical significance of the antenna is analyzed by eigenvector, characteristic angle, and modal significance, which is referred to as CMC performance matrices. The proposed antenna design and its performance improved by CMA without applying any excitation. After that, these results were cross-verified by applying feed excitation, which is called time-domain analysis. By applying the systematic design approach step by step, the overall bandwidth is improved and MIMO performance metrics are also improved. The UWB-MIMO antenna was designed with a systematic approach using CMA in Computer Simulation Tool (CST, Ver. 2018) Multilayer solver or Integral Equation solver. The Evolution of the step by step design process was performed using a systematic method and is depicted in Figure 3.

**Figure 4:**
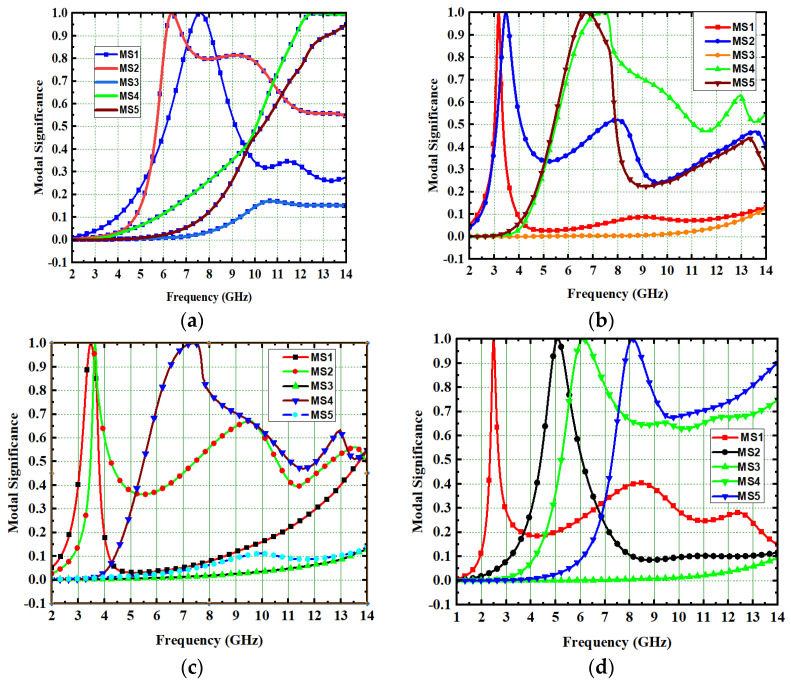
The modal significance of 2 × 2 UWB MIMO antenna. (**a**) A#0, (**b**) A#1, (**c**) A#2, (**d**) A#3.

**Figure 5:**
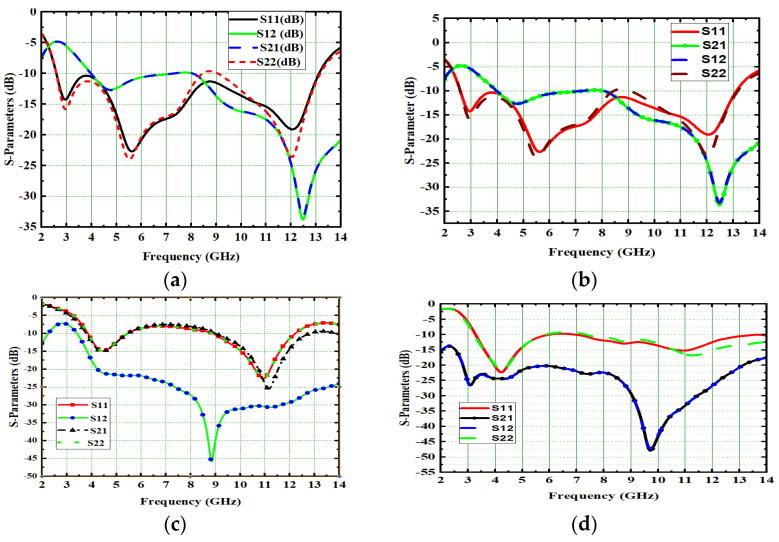
S-parameter of proposed 2 × 2 UWB MIMO antenna. (**a**) A#0, (**b**) A#1, (**c**) A#2, (**d**) A#3.

Generally, the modal significance of these four antennas, A#0, A#1, A#2, and A#3, are evaluated without applying any feed using CMA, as shown in Figure 4. The same four antennas, A#0, A#1, A#2, and A#3, were analyzed using time-domain analysis by applying feed and excitation; the associated S-parameters are depicted in Figure 5. Initially, the high-compact 2 × 2 antenna was constructed with two circular patch monopoles and was composed with rectangular discs without a ground plane, referred to as A#0. The A#0 was analyzed by generating characteristic modes. In A#0, almost five characteristic modes were generated to analyze physical insight into the structure of the antenna, and associated modal significance are depicted in Figure 4a. The characteristics modes 1, 2, 4, and 5 contribute to achieving a higher bandwidth. The CM3 did not contribute anything to achieve wideband. The modal significances of A#1, A#2, and A#3 are depicted in Figure 4b–d. The associated S-parameters of A#0, A#1, A#2, and A#3 are shown in Figure 5a–d.

**Figure 6:**
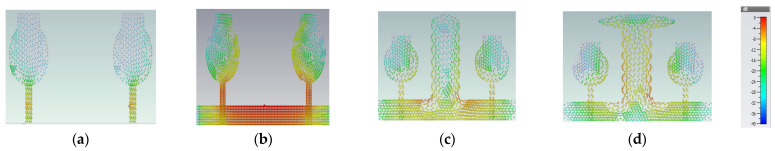
The fundamental CMC effect in the evolution of the 2 × 2 UWB-MIMO.

The antenna offers low isolation at low frequencies, but it is also quite good at the remaining frequencies in the entire UWB spectrum, as observed from its S-parameters. In the design evolution of the high-compact 2 × 2 MIMO antenna, the fundamental Characteristic Mode Current (CMC) changes the direction of interaction with MIMO elements, as depicted in Figure 6.

Figure 6 explains the CMs of CMC in A#0, A#1, A#2, and A#3, respectively. The fundamental CM1 of CMC interacts with one monopole rather than the ground plane to the monopole because A#0 is not in contact with the ground. Hence, greater interference is generated between the two radiating monopoles in A#0, which consequently increases the mutual coupling. The corresponding current distributions of A#0 in CM1is depicted in Figure 6a. When CMA is applied to the A#0, it generates various CMs, each with its own modal significance, eigenvalues, and characteristic angle. The eigenvalue represents the effectiveness of a mode, while the modal significance represents relative bandwidth. The characteristic angle denotes the current distribution’s compactness at that frequency.

When compared to other CMs, the modal significance closest to unity (M_s_ = 1) indicates that the mode resonates and is most effective at that frequency. The eigenvalue reaches zero (λ_n_ = 0) when that mode of operation is resonated at that frequency. The characteristic angle where it crosses 180° (θc = 180°) is where the corresponding frequency resonates at that frequency.

**Figure 7:**
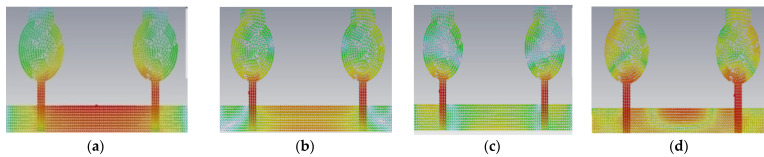
The A#1 surface current densities at (**a**) 3.8 GHz, (**b**) 6.8 GHz, (**c**) 9.8 GHz, (**d**) 12.8 GHz.

In comparison to other modes, modal significance reaches one at A#0, characteristic angle crosses 180°, and eigenvalue is zero for CM1, indicating that this CM1 resonates at this frequency and is more effective in the low-frequency range. The A#1 is composed of partial ground, and its current densities are at different frequencies in the entire operating frequency spectrum, as depicted in Figure 7.

A#1 is obtained by adding the ground plane to A#0. Therefore, all CMs are moved toward the lower frequency region, except CM1. Most of the current distributions and currents are now moving from the monopole to the ground plane of the MIMO, i.e., low interference is achieved between one monopole radiator to another monopole radiator. The modal significance of A#1 is depicted in Figure 4b. The corresponding A#1 S-parameters are shown in Figure 5b. The radiation properties at high frequencies of A#1 are enhanced compared to A#0, i.e., the bandwidth is increasing towards high frequencies. However, due to the high interacting currents between radiating elements, good isolation is not achieved at low frequencies. 

The design evolution process of the proposed antenna is executed via four (4) different design steps. Wideband frequency response was achieved by adjusting the CMs in the high-frequency region, at the high-frequency band, but a good impedance bandwidth was not achieved. Therefore, for this purpose, an I-shaped stub has been embedded in between the radiators to enhance the isolation of the elements. The design process of the I-shaped stub is explained in the following sections. The next stage of evolution is A#2.

### 2.2. Effect of Decoupling Stub

The various types of decoupling techniques, such as F-shaped stubs, Y-shaped stubs, and T-shaped stubs, are used to reduce mutual coupling [32,33,34]. The decoupling stubs are placed in between radiating elements to enhance isolation in the MIMO antenna. In this design evolution process of antenna, an I-shaped decoupling stub is combined with A#1 to form A#2. By adding an I-shaped stub to A#1, further mutual coupling is reduced. The dimensions of an I-shape stub are listed in Table 1 and are formed by stacking seven ellipses one on top of the other. The A#2 is analyzed with CMA without applying excitation. Due to the insertion of the I-shaped stub in A#2, all modes are shifted from the high-frequency band to the mid-frequency band, except CM1, hence increasing the bandwidth and isolation between radiating elements. The respective modal significance is depicted in Figure 4c.

**Figure 8:**
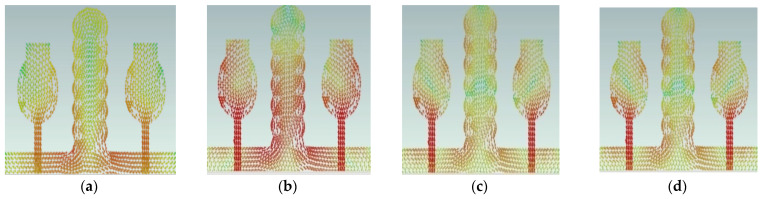
The characteristic currents of CM1in A#2 at (**a**) 3 GHz, (**b**) 6 GHz, (**c**) 9 GHz, and (**d**) 12 GHz.

The current distributions in A#2 interact with the monopoles, I–shaped stub, and the ground plane. Earlier, the scattered currents interacted from monopoles to monopoles, and monopoles to the ground. Now the currents are moving from stubs to the ground and from monopoles to the ground, so mutual coupling is reduced between radiating elements. The current distributions of A#2 at different frequencies are depicted in Figure 8.

The A#2 is analyzed by applying feed with excitation in time-domain analysis. The respective S-parameters are shown in Figure 5c. Figure 5c explains that the isolation of the monopole radiators is increased. However, mutual interaction between the elements is still observed at some frequencies that will degrade the performance matrices of MIMO, such as CCL, MEG, ECC, and DG. By placing I-shaped decoupling stubs in MIMO, they reduce the mutual coupling, but not to a higher extent.

Therefore, we need to enhance further isolation. For that purpose, we added an elliptical patch at the top of the I-shaped stub, and it becomes a T-shaped stub. The resultant antenna is called A#3. Characteristic Mode Analysis is applied to A#3 to generate the respective five characteristic modes. By adding an elliptical patch to the I–shaped stub, most of the characteristic modes are shifted from the high-frequency region to the mid-band frequency spectrum [35], which results in an increase in the bandwidth over the UWB frequency spectrum. The corresponding modal significances are shown in Figure 4d. The time-domain analysis is applied to A#3 with feed and excitation. The respective S-parameters are observed and depicted in Figure 5d. The scattering fields and current distributions are moving among radiating elements, stubs, and the ground plane, instead of moving from radiator to radiator. Hence, high isolation is achieved in this antenna.

**Figure 9:**
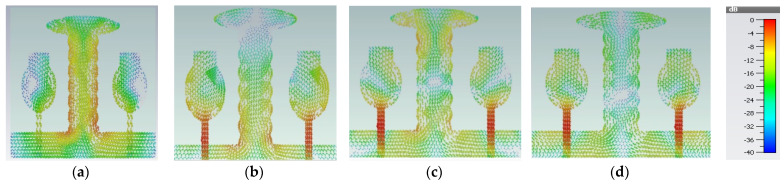
The effect of characteristic currents due to CM1 in A#3 at (**a**) 3 GHz, (**b**) 4 GHz, (**c**) 5 GHz, and (**d**) 6 GHz.

**Figure 10:**
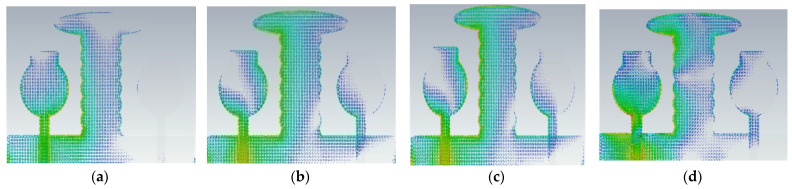
Current densities due to CM1 in A#3 at (**a**) 3 GHz, (**b**) 4 GHz, (**c**) 5 GHz, and (**d**) 6 GHz.

The time-domain analysis is applied to A#3 with feed and excitation. The respective S-parameters are depicted in Figure 5d. By integrating elliptical patch on to an I-shaped decoupling stub, the isolation of the MIMO antenna is improved, and good performance matrices are achieved. At various frequencies, the A#3 characteristic currents and current densities are simulated and shown in Figure 9 and Figure 10, respectively [36,37,38].

## 3. Results of High-Compact 2 × 2 UWB-MIMO Antenna

**Figure 11:**
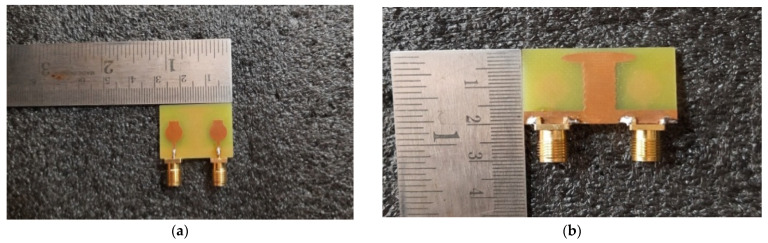
The Proposed 2 × 2 UWB-MIMO fabricated antenna (**a**) Front View (**b**) Back View.

**Figure 12:**
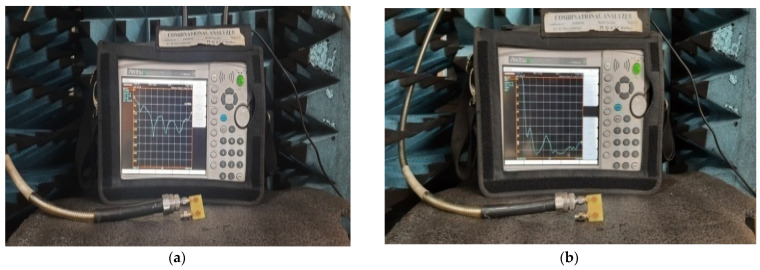
Prototype of the proposed 2 × 2 UWB-MIMO antenna. (**a**) Return Loss, (**b**) Isolation.

**Figure 13:**
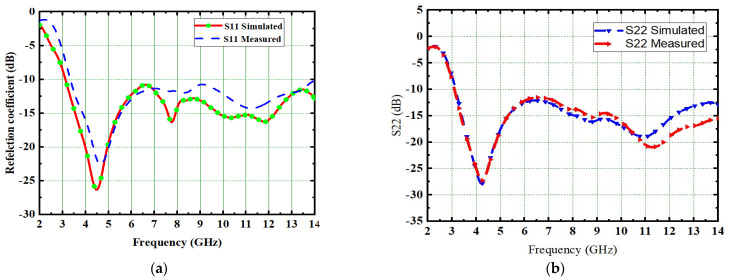
Comparisons of simulated and measured (**a**) Reflection coefficient (S11) (**b**) S22.

**Figure 14:**
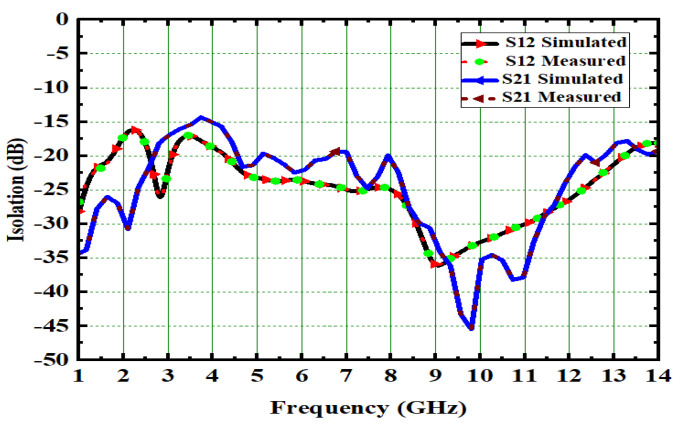
Isolation in 2 × 2 UWB-MIMO Antenna.

The performance of the designed 2 × 2 antenna was measured and found good isolation from 3.1 GHz to 13.25 GHz of the entire frequency spectrum, with more than 23 dB isolation and a gain of 4.2 dB. The radiation efficiency and fractional impedance bandwidth is 82.5% and 124.15%, respectively. The fabricated compact UWB MIMO 2 × 2 is depicted in Figure 11. The return losses of an antenna are found in S_11_ ≤ 10 dB of the entire UWB spectrum. Isolation is less than 23 dB and is depicted in Figure 12. Figure 13 represents a comparison of tested and simulated values of S_11_ and S_22_. Figure 14 represented the comparisons of simulated and tested values of isolation between the elements of 2 × 2 UWB-MIMO Antenna.

**Figure 15:**
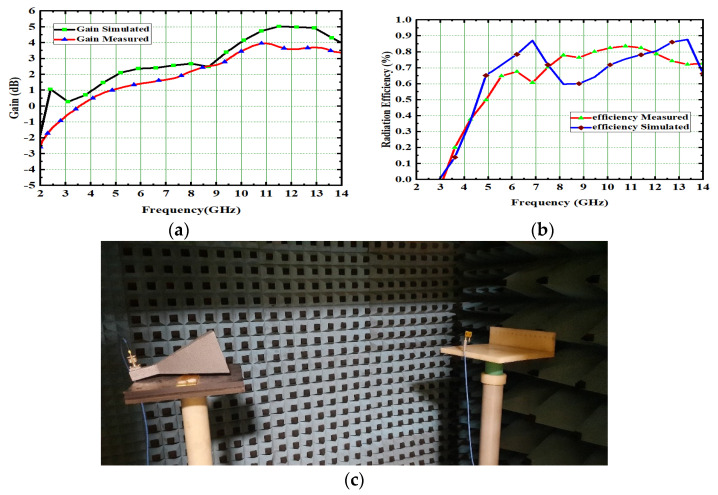
(**a**) Gains and (**b**) Radiation efficiency, (**c**) Gain measurement setup.

**Figure 16:**
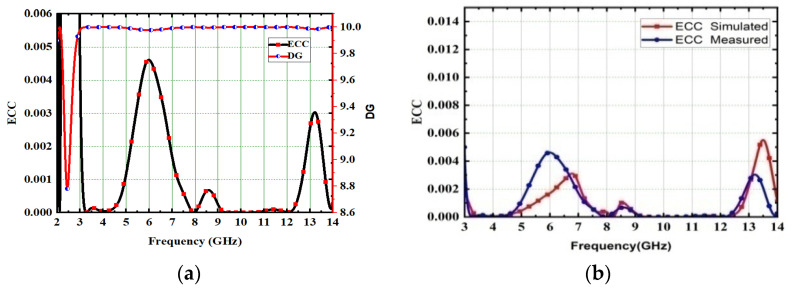
(**a**) DG with ECC, (**b**) ECC Measured.

**Figure 17:**
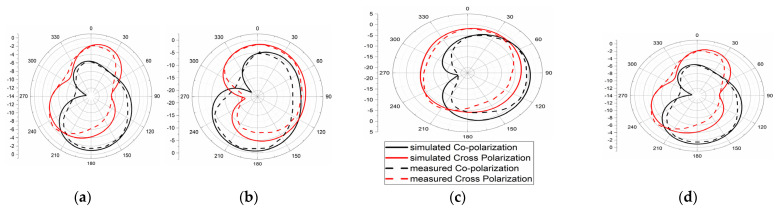
The radiation Pattern of E-Plane (**a**) 4.5 GHz and (**b**) 6.8 GHz, and H-plane at (**c**) 4.5 GHz and (**d**) 6.8 GHz.

The overall performance of MIMO depends on its gain and efficiency. By using a sophisticated step by step design procedure, this antenna achieved 4.2 dB gain and 82.5% radiation efficiency, as depicted in Figure 15. The measurement of gain and its setup is depicted in Figure 15c. The key performance of MIMO antennas is diversity performance, and antenna designers focus on diversity characteristic parameters while designing UWB-MIMO antennas to meet the challenges in wireless communication. One of the most significant parameters of the diversity parameter is the ECC, which shows how the radiating elements interact with each other when placed very close together in MIMO. The respective ECC is depicted in Figure 16b, and its value is 0.005. Diversity gain is measured, and its value is 9.89, as shown in Figure 16a. The E-pattern and H-pattern are tested, and simulated results are depicted in Figure 17 at frequencies of 4.5 GHz and 6.8 GHz, respectively.

Still, there is a great need to achieve high isolation and ECC for reliable wireless communication. Therefore, we can extend this 2 × 2 UWB-MIMO antenna into a 4 × 4 UWB-MIMO antenna to achieve good diversity performance using Characteristic Mode Analysis.

## 4. 4 × 4. UWB-MIMO Antenna Design

The designed compact 2 × 2 UWB-MIMO antenna has dimensions of 0.29 λ_0_ × 0.21 λ_0_ (28 × 20 mm^2^), and it is extended into a 4 × 4 high-compact UWB-MIMO antenna with dimensions of 0.290 × 0.420 (28 × 40 mm^2^) printed on FR-4 having $\varepsilon_{r}\;$= 4.3 with tanẟ = 0.002.

**Figure 18:**
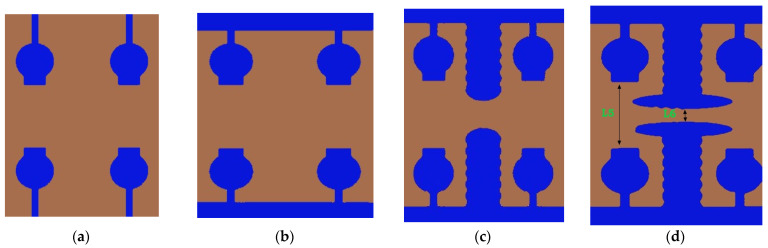
The Evolution of 4 × 4 UWB MIMO antenna design. (**a**) A#0, (**b**) A#1, (**c**) A#2, (**d**) A#3.

**Figure 19:**
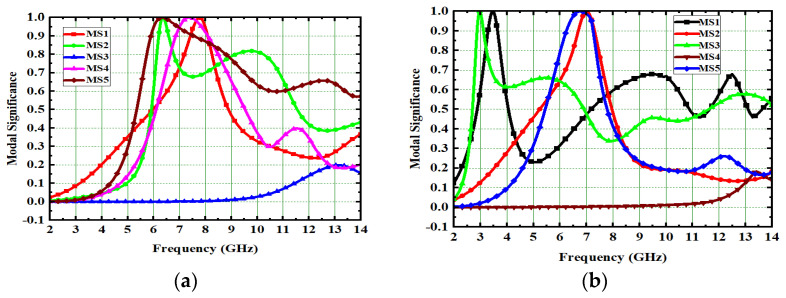

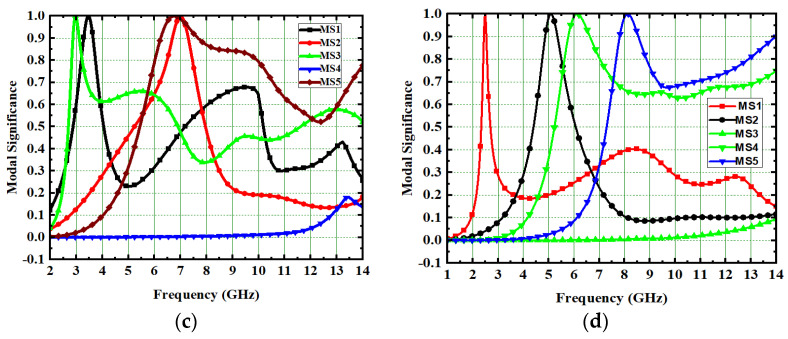
Modal significance of 4 × 4 antenna. (**a**) A#0, (**b**) A#1, (**c**) A#2, (**d**) A#3.

**Figure 20:**
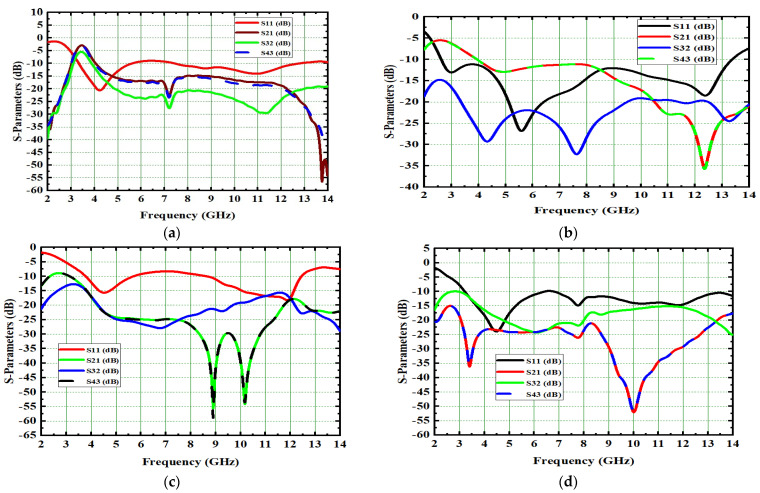
S-parameter of 4 × 4 antenna. (**a**) A#0, (**b**) A#1, (**c**) A#2, (**d**) A#3.

This 4 × 4 UWB-MIMO design process is performed in stages denoted as A#0, A#1, A#2, and A#3, respectively. The 4 × 4 UWB-MIMO systematic design process is depicted in Figure 18. Increasing the elements in MIMO will increase diversity performance. The antenna is designed without the ground plane and applies characteristic modes to observe its modal significance, as seen in the corresponding graphs shown in Figure 19a. Figure 20a explains the S-parameters of the above A#0. The CMs are good at high frequencies but not at low frequencies. A ground plane is added to A#0, along with a decoupling stub to form A#1, A#2, and A#3; the corresponding modal significance and S-parameters are depicted in Figure 19b–d and Figure 20b–d.

**Figure 21:**
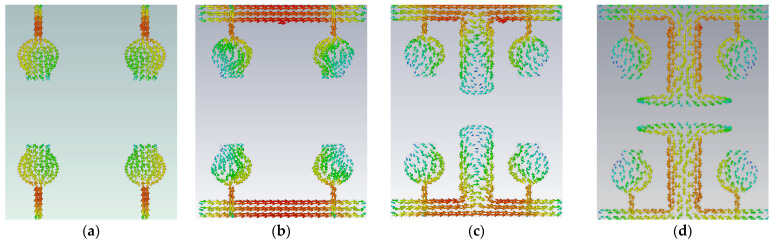
The characteristic mode currents in the design process of high-compact 4 × 4 UWB-MIMO antenna. Characteristic current effect due to CM1 of A#3 at (**a**) 3 GHz (**b**) 4 GHz (**c**) 5 GHz (**d**) 6 GHz.

**Figure 22:**
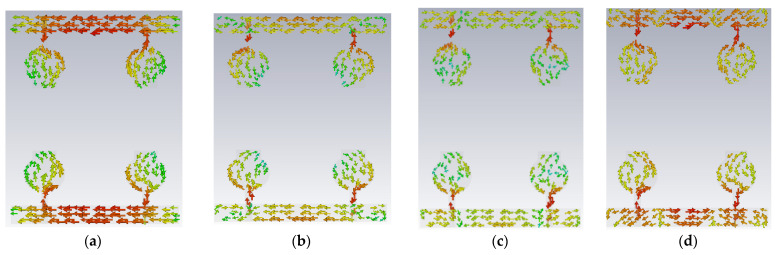
The surface current densities of A#1 at (**a**) 3.8 GHz, (**b**) 6.8 GHz, (**c**) 9.8 GHz, and (**d**) 12.8 GHz.

**Figure 23:**
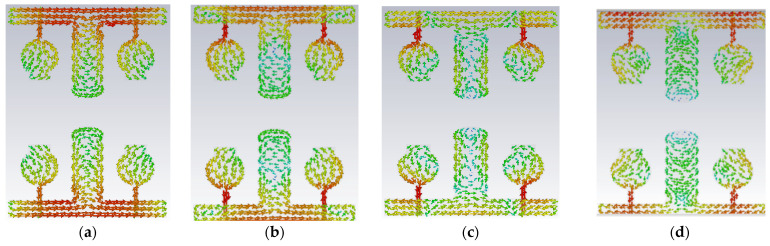
The effect of characteristic mode current in A#2 (**a**) 3 GHz (**b**) 6 GHz (**c**) 9 GHz (**d**) 12 GHz.

**Figure 24:**
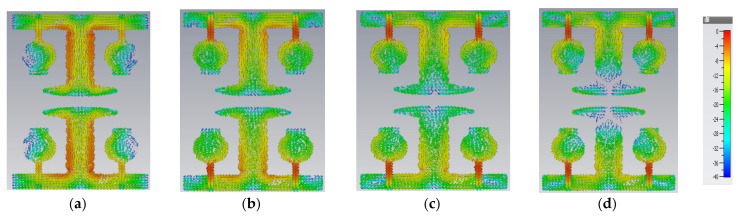
A#3 Characteristic current effect due to CM1 at (**a**) 3 GHz (**b**) 4 GHz (**c**) 5 GHz (**d**) 6 GHz.

**Figure 25:**
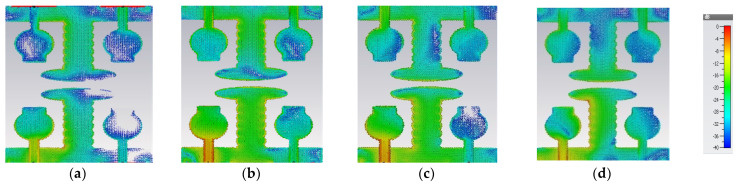
Surface current density of A#3. (**a**) 3 GHz, (**b**) 4 GHz, (**c**) 5 GHz, (**d**) 6 GHz.

A#0 is designed without a ground plane, A#1 has a partial ground plane, A#2 is made up of an I-shaped stub, and A#3 is made up of an elliptical stub. The fundamental characteristics of currents and the distribution of CM1 are depicted in Figure 21. Adding stubs to a 4 × 4 antenna is similar to adding stubs to a 2 × 2 antenna. The surface current density and its distributions of A#1 at various frequencies are shown in Figure 22. By adding an I-shaped stub to A#1, it becomes A#2. The A#2 characteristic currents and distributions of CM1 are expressed at various frequencies, as depicted in Figure 23. For A#3, the characteristic current effect due to CM1 and current densities at different frequencies are depicted in Figure 24 and Figure 25, respectively.

## 5. Results of High-Compact 4 × 4 UWB-MIMO Antenna

**Figure 26:**
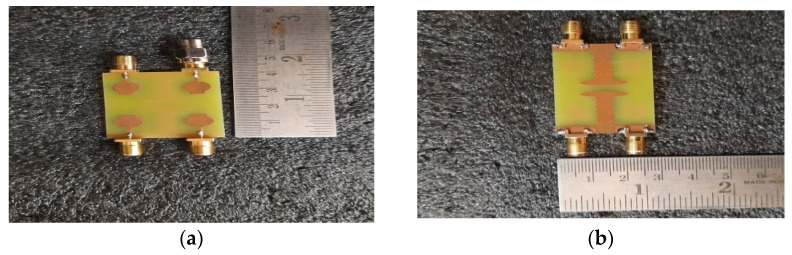
High-compact 4 × 4 UWB-MIMO antenna fabricated prototype. (**a**) Top View, (**b**) Bottom View.

**Figure 27:**
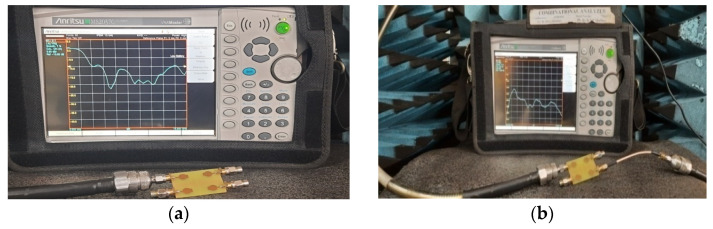
High-compact 4 × 4 UWB-MIMO Antenna Measurement. (**a**) S_11_, (**b**) Isolation.

**Figure 28:**
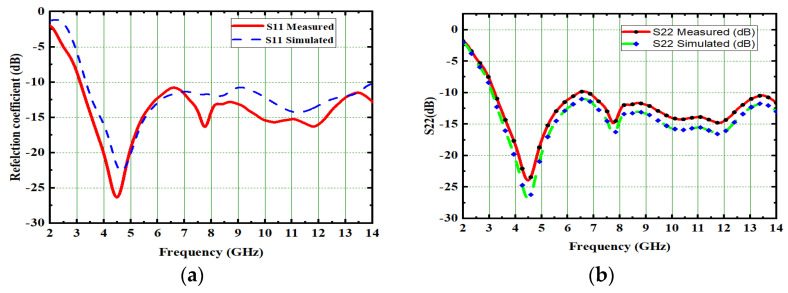
Comparison of simulated and measured (**a**) S_11_ and (**b**) S_22_.

The performance of the MIMO depends on its reflection coefficient and isolation among the MIMO elements. Good isolation is achieved through a systematic design approach called Characteristic Mode Analysis. The designed antenna covers 3.1 GHz–13.75 GHz at a bandwidth of 10.65 GHz. The prototype of 4 × 4 is depicted in Figure 26. The snapshot of the measured reflection coefficients and isolation using VNA are shown in Figure 27. The simulated values of S_11_ and S_22_ are compared with tested values, as depicted in Figure 28. Both the results are in acceptable agreement, as displayed and achieved with the help of CMA. The measured reflection coefficients and isolation are ≤10 dB and ≤25 dB, respectively.

**Figure 29:**
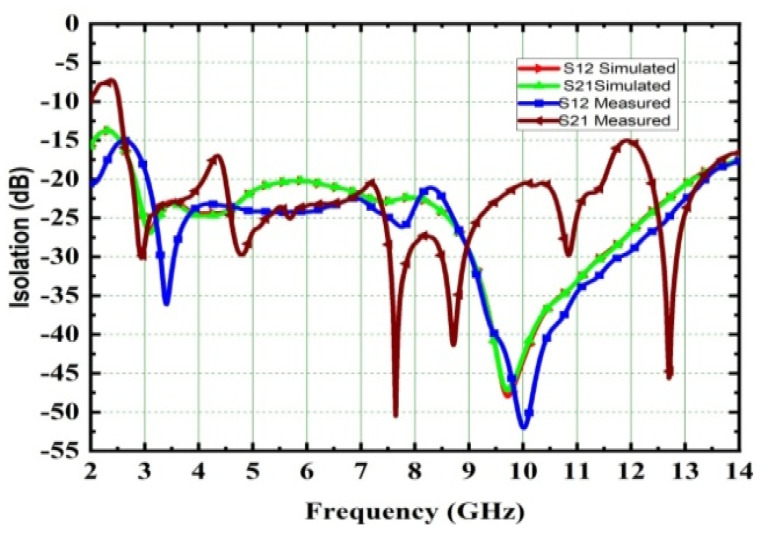
Isolation of 4 × 4 UWM-MIMO antenna.

**Figure 30:**
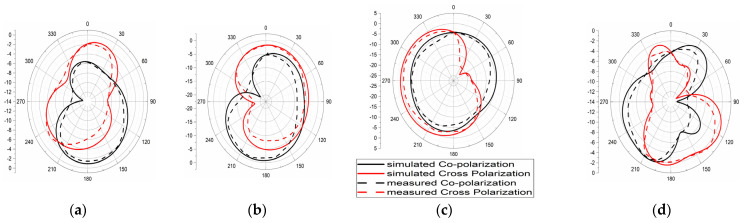
The radiation pattern of E-plane at (**a**) 4.5 GHz and (**b**) 6.8 GHz, and H-plane at (**c**) 4.5 GHz and (**d**) 6.8 GHz.

Reduced mutual coupling, high isolation is achieved among all ports; its value is more than 25 dB and it is depicted in Figure 29. The Fields of the 4 × 4 MIMO at 4.5 GHz and 6.8 GHz were measured and are depicted in Figure 30. The E- and H-plane radiation patterns of the radiator are obtained by applying excitation at port one while the remaining ports are matched with a 50Ω load. The obtained radiation patterns are similar for radiator 2, radiator 3, and radiator4 at respective ports 2, 3, and 4. With the help of DRH20, the radiation pattern is measured in an anechoic chamber. The pattern in the H-plane would be almost omni-directional, whereas the pattern in the E-plane is bi-directional.

### 5.1. 4 × 4 MIMO Gain and Radiation Efficiency

**Figure 31:**
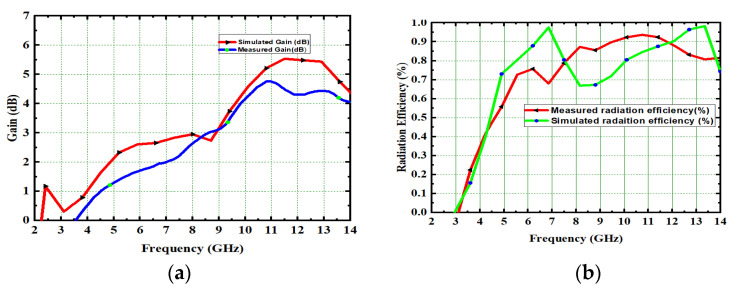
High-compact 4 × 4 UWB-MIMO antenna. (**a**) Gain, (**b**) Radiation efficiency.

The gain and radiation efficiency of a high-compact 4 × 4 UWB-MIMO antenna is 4.8 dB and 92.2%, respectively. The high-compact proposed 4 × 4 UWB-MIMO antenna radiation, when efficiency simulated and measured, varies from 92.2% to 94% and 90.2% to 92.2%, respectively. These measured values are slightly different from simulated values because of connector losses and fabrication losses in the antenna. The gain and radiation efficiency of the designed high-compact 4 × 4 MIMO antenna is depicted in Figure 31.

### 5.2. Diversity Parameters

The ECC, DG, MEG, and CCL are known as the diversity parameters of the MIMO antenna [37,38,39]. These parameters should be in the acceptable range only, otherwise the performance of MIMO is not good and is therefore unsuitable for reliable wireless communication. Therefore, the above diversity parameters are calculated and plotted with the help of MATLAB code, and they are also verified with measured values.

#### 5.2.1. ECC and DG

One of the key performances of MIMO antennas is ECC. The acceptable ECC value in MIMO is below 0.5, but by using a systematic design process with the CMA technique, the proposed high-compact 4 × 4 UWB-MIMO antenna achieved a 0.0045 value across the entire UWB spectrum. The ECC explains how the radiating elements interact with each other. No interaction between the radiating elements means that ECC is 0. The ECC is calculated from S-parameters [37].

(4) $$\mathrm{ECC}=\frac{{\left|S_{ii}{}^{*\;}S_{ij}+S_{ji}{}^{*}S_{jj}\right|}^{2}}{\left(1-\left({\left|S_{ii}\right|}^{2}+{\left|S_{ji}\right|}^{2}\right)\right)\left(1-\left({\left|S_{jj}\right|}^{2}+{\left|S_{ij}\right|}^{2}\right)\right)}\;$$

where *S_ii_*, *S_ij_*, *S_ji_*, and *S_jj_* are the S-parameters of UWB-MIMO. This Equation (4) is correct for a 2 × 2 antenna. If MIMO has more than two radiating elements, it is better to calculate ECC with the help of Avg powers along with its directions. The ECC is also represented with the symbol ρ. This represents how the radiating elements are correlated with each other. It measures how the *i*th radiating element is correlated with the *j*th element in the designed MIMO antenna. The method of calculating ECC using S-parameters is not advisable because most of the microstrip antennas used by planners and printers are lossy. Therefore, we can calculate ECC from far-field [38] radiation patterns.

(5) $$\rho_{\mathrm{ij}}=\frac{|\int_{0}^{2\pi }\int_{0}^{\pi }[\mathrm{XPR}.E_{\theta i}E_{\theta j}^{*}P_{\theta }+E_{\Phi i}E_{\Phi j}^{*}P_{\Phi }]d\Omega {|}^{2}}{\int_{0}^{2\pi }\int_{0}^{\pi }[\left[\mathrm{XPR}.E_{\theta i}E_{\theta i}^{*}+E_{\Phi i}E_{\Phi i}^{*}P_{\Phi }\right]d\Omega X\int_{0}^{2\pi }\int_{0}^{\pi }{\mathrm{XPRE}}_{\theta j}E_{\theta j}^{*}P_{\theta }+E_{\Phi J}E_{\Phi J}^{*}P_{\Phi }]d\Omega }$$

XPR is cross-polarization rate (XPR = Pv/P_H_). The first port is fed with the signal, whereas all remaining ports are terminated and matched with a 50 Ω load. The impact of fading can be reduced by composing the antenna with several radiating elements using a diversity technique. The DG is also an important diversity parameter. The DG is also expressed [37] in terms of ECC.

(6) $$DG=10\sqrt{1-ECC^{2}}$$

**Figure 32:**
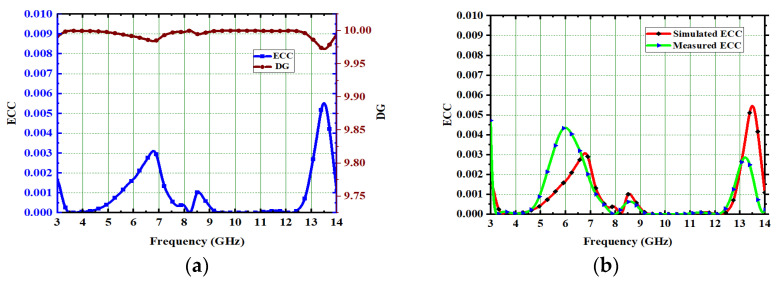
(**a**) ECC with DG, (**b**) ECC without DG.

The ECC and DG measured and simulated values are plotted and depicted in Figure 32. Here, the ECC is 0.0045, achieved for the proposed antenna; this is a very low value and indicates the low correlation between the MIMO elements. The acceptable value of DG in MIMO is 9.59 dB. The proposed high-compact 4 × 4 UWB-MIMO antenna achieved more than 9.982. The low ECC and high isolation in the MIMO system increase the reliability of the communication system.

#### 5.2.2. MEG

**Figure 33:**
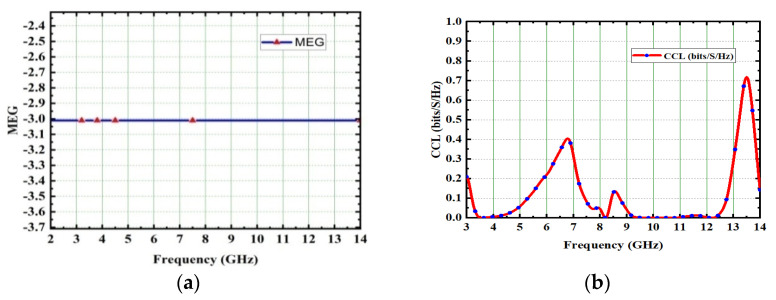
(**a**) MEG, (**b**) CCL.

One of the performance matrices of an MIMO antenna is the MEG [38]. The MEG is expressed as:

(7) $$MEG=\int_{0}^{2\pi }\int_{0}^{\pi }[\frac{XPR}{1+XPR}F_{\alpha }\left(\alpha ,\beta \right)P_{\alpha }\left(\alpha ,\beta \right)+\frac{1}{1+XPR}F_{\beta }\left(\alpha ,\beta \right)P_{\beta \Phi }\left(\alpha ,\beta \right)]\sin\alpha d\alpha d\beta \;$$

where *F_𝛼_* and *F_β_* are patterns of power in the MIMO antenna system, respectively. Generally, in the MIMO antenna system, the MEG between two ports is always less than −3 dB. In the proposed 4 × 4 antenna, the MEG measured between port 1 and port 3 is −3.1 dB, as depicted in Figure 33a.

#### 5.2.3. The Channel Capacity Loss

The CCL matrices are an important indicator of radiation performance in the MIMO antenna system. The minimum and acceptable limit of CCL in the MIMO system is 0.4 bits/s/Hz. It is mathematically defined as [39]:

Closs = −log_2_det(Y^R^) (8)

(9) $$Y=\left[\begin{array}{cccc} Y11 & Y12 & Y13 & Y14\\ Y21 & Y22 & Y23 & Y24\\ Y31 & Y32 & Y33 & Y34\\ Y41 & Y42 & Y43 & Y44 \end{array}\right]$$

and also expressed as:

(10) $$Y_{\mathrm{nm}=-\left(S_{\mathrm{nn}}^{*}S_{\mathrm{nm}}+S_{\mathrm{mn}}^{*}S_{\mathrm{nm}}\right)}$$

Here n, m = 1 to 4. The simulated and examined values of CCL are included in Figure 33b. Over the entire UWB frequency spectrum, the designed antenna achieved a CCL of 0.399 bits/s/Hz.

## 6. Comparisons of Proposed Work with other Models

The performance parameters of the proposed UWB-MIMO antennas are compared with existing UWB-MIMO antenna working models and discussed in the literature, and they are shown in Table 2. The performances of UWB-MIMO antennas were compared, taking into consideration parameters such as the compactness of the antenna, i.e., size and area, design approach, isolation parameters, ECC, channel capacity loss, and MEG, as shown in Table 2. The major advantages of this proposed antenna are good isolation, bandwidth, and the low ECC achieved using CMA.

## 7. Conclusions

The design of compact stub-based UWB-MIMO antennas (2 × 2 and 4 × 4 antennas) are carried out using Characteristic Mode Analysis. Using CMA, a systemic design approach has been achieved. The proposed 2 × 2 and 4 × 4 UWB-MIMO antennas achieve a high isolation of 23 dB and 25 dB, and their bandwidths are 10.11 GHz and 10.775 GHz, respectively. The IBW of compact 2 × 2 and 4 × 4 UWB-MIMO is 124.15% and 126.40%, respectively. The diversity parameters of 2 × 2 and 4 × 4 UWB-MIMO antennas, such as ECC, CCL, MEG, and DG, are 0.005 and 0.0045, 0.39 and 0.39, −3.1 dBi and −3.11 dBi, and 9.978 and 9.982, respectively. The overall performance of both 2 × 2 and 4 × 4 antennas meet the requirements of wireless communication.

## Figures and Tables

**Table 1:** The designed parameters (Units in mm).

L1	L2	L3	d1	W1	W2
2	7	12.5	17.5	4	1.2
L4	r1	r2	r3	r4	r5
3.4	3	2	3	2	7
r6	Ws	W_L_	Wh	hp	L5
1.735	20	20 & 40	1.4	0.035	8
L6	r				
8	3.6				

**Table 2:** Comparative Analysis with Literature.

Ref.	Design Methodology	Dimensions (mm^2^)	Isolation (dB)	ECC	CCL (b/s/Hz)
[2]	Inverted L-shaped strip	60 × 30	34	0.002	0.5
[3]	Decoupling stubs in hexagonal shape	34 × 20	20	0.2	---
[4]	I-shaped decoupling stub	25 × 25	17	0.01	---
[6]	Fractal slot and flipped in horizontal	53 × 35	19	0.007	---
[7]	cut in substrate and placed orthogonal	58 × 28	20	0.008	---
[8]	U-Shaped with T-shaped decoupling stub	30 × 45	29.5	0.00185	---
[19]	Pattern diversity with CMT	85 × 50	20	0.03	---
[23]	CSRR and decoupling stub inverted L-shape	23 × 29	15	0.15	---
[30]	T-shaped Stub	18 × 36 × 18.5	20	0.02	---
[32]	Space diversity	60 × 60	16	0.005	---
[35]	Characteristic Mode Analysis (CMA) with vias and fork-shaped stubs	20 × 28	23	0.005	---
Prop (2 × 2)	CMA with T-shaped decoupling Stub	28 × 20 × 1.6	23	0.005	0.029
[5]	Placement in orthogonal form	38 × 38	20	0.01	0.4
[9]	Polarized diversity with defected ground system	38.3 × 38.3	17	0.02	---
[10]	Fractal slot and flipped in horizontal	40 × 40	12	0.07	0.4
[32]	Space diversity and pattern diversity	90 × 60	20	0.005	---
[35]	Characteristic Mode Analysis (CM) with vias and Fork-shaped stubs	40 × 28	17	0.01	---
[39]	Parasitic decoupler	60 × 60 × 1.52	21	0.001	--
Prop (4 × 4)	CMA with T-shaped decoupling Stub	28 × 40 × 1.6	25	0.0045	0.39

## Data Availability

The data presented in this research are available on request from the corresponding author.
